# Emotional analysis of multiplayer online battle arena games addiction

**DOI:** 10.3389/fpsyg.2024.1347949

**Published:** 2024-05-09

**Authors:** Enwu Huang, Yalong Xing, Xiaozhou Song

**Affiliations:** ^1^School of Design, Fujian University of Technology, Fujian, China; ^2^Faculty of Innovation and Design, City University of Macau, Macau, Macao SAR, China; ^3^School of Humanities, Fujian University of Technology, Fujian, China

**Keywords:** games addiction, PLS-SEM, MOBA games, emotional design, leisure theory, uses and gratification

## Abstract

**Introduction:**

Multiplayer Online Battle Arena (MOBA) games have garnered widespread popularity as a form of recreational activity. The launch of League of Legends (LoL), a prominent MOBA game, has captivated the enthusiastic pursuit of gamers in the MOBA community. The surge in MOBA game fervor, coupled with the influence of personal emotions, can result in excessive engagement, ultimately leading to addiction.

**Objective:**

This study aimed to investigate the moderating effects of visceral perception, behavior, and reflection on game players’ addiction within the framework of Leisure Theory (LT), Uses and Gratification Theory (UGT), and Emotional Design Theory (EDT).

**Methods:**

A hypothesized theoretical model was developed and empirically evaluated based on 236 self-reported validated responses from MOBA gamers. SPSS (version 26) was employed for demographic analysis and game duration analysis. The measurement model and structural model analyses were conducted in two stages using Partial Least Squares Structural Equation Modeling (PLS-SEM) with SmartPLS 4.1.0 to validate the nine theoretical hypotheses.

**Results:**

It has been observed that personal emotions significantly contributes to MOBA game addiction during gamers’ leisure time or moments of gratification. Specifically, a noteworthy connection exists between two dimensions, namely gamers’ behavior and reflection, demonstrating a positive correlation with gaming addiction. Without taking entertainment as a motivating factor, there is no significant relationship between gamers’ leisure-time and visceral perception.

**Conclusion:**

This study enhances the theoretical model of gamers’ behavioral motives in engaging with MOBA gaming and contributes to the expansion of research on game addiction theory. These findings offer valuable theoretical insights for emotional design in games and the design of mechanisms for preventing game addiction.

## Introduction

Video gaming has emerged as one of the most popular recreational activities globally (Griffiths et al., 2012; Pornpongtechavanich et al., 2022). Initially perceived as a mild leisure pursuit, video games have been demonstrated to possess addictive features, particularly in strategy video games like multiplayer online battle arena (MOBA) (Nuyens et al., 2016; Han et al., 2020). MOBA games constitute a significant segment of the rapidly expanding and captivating eSports genre, closely intertwined with players’ social experiences (Wang et al., 2023). This connection has led to widespread criticism of the genre, citing perceived adverse effects on players’ physical and psychological well-being, lifestyle, and work-study balance (T’ng et al., 2023). However, recent pro-game research on the genre has challenged these assumptions, highlighting MOBA games for their potential to foster cultural inclusiveness, enhance social connectedness, alleviate stress, boost cognitive skills, and facilitate skill transferability (Mora-Cantallops and Sicilia, 2018; Wang et al., 2023).

In 2021, Tencent officially launched the League of Legends (LoL) MOBA game in China, generating significant excitement among Chinese gaming enthusiasts (Ye, 2021). The integration of MOBA games with LoL PC players led to a surge in the MOBA game industry, further fueled by LoL’s recognition as an official medal sport at the Asian Games Hangzhou in 2022 (see official website). However, these developments heightened concerns among parents and other stakeholders about potential issues arising from the escalating popularity of this gaming craze (Lieberoth and Fiskaali, 2021). Empirical studies have identified various factors contributing to gaming addiction (Nuyens et al., 2016; Xia et al., 2019; Lieberoth and Fiskaali, 2021; Wut et al., 2021). MOBA games, such as LoL and Honor of Kings (HOK) in China, in particular, have gained notoriety for their adverse effects on gamers, including user retention problems (Wang et al., 2023) and the promotion of gaming disorder through frustration-induced continuance (T’ng et al., 2023). To address and manage gaming disorder more effectively, researchers have proposed theoretical models based on diverse perspectives and measurement methods.

Many studies have explored the personality characteristics of MOBA game players, their post-game gratification behaviors, and motivations for continued gaming. One study, drawing on communication theory, posited that not all MOBA game behaviors are consciously motivated (LaRose and Eastin, 2004). Recurrent behavior in stable environments can lead to habit formation, reducing the cognitive load associated with decision-making, and transforming outcome expectations into habits (De Grove et al., 2016). Another study, utilizing the Five Factor Model, maintained that MOBA game players’ traits of extraversion, agreeableness, and openness interact with in-game performance, affecting player behavior (Matuszewski et al., 2020).

Based on the premise, the behavior of MOBA gamers appears to be related to their personality, manifesting as various individual characteristics that can influence thinking and behaviors rooted in attitude, motivation, needs, and emotions, thereby impacting their external or internal behaviors (Wang and Yu, 2017). Furthermore, the gratification of MOBA game players with their in-game performance can unveil the correlation between emotional needs and behavioral intentions (Wut et al., 2021). Experimental and self-report measures have indicated that players display impulsiveness during MOBA gameplay, and this impulsiveness serves as a crucial indicator for identifying excessive involvement in MOBA games (Nuyens et al., 2016).

However, in advancing a tentative theory regarding eSports skills, Larsen (2020) employed a three-pronged approach involving discussion, reflection, and evaluations of players who watched 100+ hours of eSports events on social media. This inferential theory not only guided and enhanced gamer skills at competitive levels but also solidified the argument that theoretical modeling, coupled with empirical research, can enhance gamers’ behavior in MOBA games. Analyzing data from game players of TI4 (The International Dota 2 Championships), Xia et al. (2019) found that tactical awareness had a greater influence than manipulation skills in team games, as evidenced by Brown-Mood tests (Brown and Mood, 1951). However, Xia and colleagues did not delve into the influence of intrinsic factors, such as tactical awareness, on individual players’ game reflection level, operational skills, and in-game performance in non-tournament MOBA games. Additionally, certain studies have highlighted indispensable influences of factors like emotion, external environment, and system. Conducting an assessment of game and players’ experience using multiple system, user, and context influence parameters, Suznjevic et al. (2019) provided references for future Quality of Experience (QoE) assessment tools to analyze reports on MOBA gamers’ self-evaluation. Building upon this assessment, Pornpongtechavanich et al. (2022) constructed a QoE model incorporating human, context, and system factors to explore the impact of MOBA game quality on players’ game perception. They scrutinized influential factors on game quality and audio-visual quality assessment, encompassing the player’s emotional state, physical and mental constitution, social background and perceptions; the external physical and social environment, service content and innovativeness; as well as the game system itself (Pornpongtechavanich et al., 2022). The model lays a theoretical foundation for further exploration of the affective analysis of MOBA game addiction.

This study contributes to the research on MOBA gaming addiction by investigating the moderating effects of visceral perception, behavior, and reflection on the variables of leisure and gratification. Furthermore, the study expands the theoretical model of game players’ behavioral motives by integrating the theories of leisure (LT), uses and gratification (UGT), and emotional design (EDT).

## Theoretical framework

### Emotional design

Understanding, expressing, and communicating emotions constitute fundamental human abilities. Integrating positive emotional elements into design significantly enhances users’ emotional experiences (Yoon et al., 2020). In his bestseller, “Emotional Design: Why We Love (or Hate) Everyday Things,” Norman (2004) introduced an innovative theory of emotional design (EDT), challenging the preceding practicality and usability paradigm. Norman hypothesized the heightened importance of the emotional aspect in design, proposing three interdependent design levels: visceral design, behavioral design, and reflective design (Norman, 2004). Norman (2004) elucidated these concepts as follows: Visceral design, preceding consciousness and cognition, lays the foundation for esthetics, appearances, and initial impressions encompassing visual and auditory senses. Behavioral design pertains to user experience, including functionality, performance, and usability, representing external expressions of user pleasure and utilitarian emotions. Reflective design involves consciousness, higher emotions, and perceptions, which vary across cultures, experiences, education, and individual differences. However, EDT elucidated the impact of emotions on responses to design objects (Zachry, 2005), spanning product, interior, and game design. Therefore, when considering emotional studies in design, the consequential themes are emotional effects on user behavior and reflection (Yoon et al., 2020), especially in the realm of human-computer interaction (Imbesi, 2010). The intricate relationship between emotional responses to design and users’ visceral, behavioral, and reflective reactions directly influences the gaming experience of MOBA players. Abbasi et al. (2023) found that visually appealing design and graphics may lead to a positive attitude toward games and the games’ visual and auditory aspects may create a sensory experience that is positively related with game engagement. Patzer et al. (2020) examined the potential for game involvement in terms of behavioral manifestations such as gamer motivation and intention. In addition, gamers’ imagination, emotions, and sensory experiences could trigger cognitive, emotional, and behavioral engagement with the game (Bojan et al., 2021). The above studies show that games are designed with cognitive and affective factors, but cognition is related to behaviors such as motivation.

Hence, this paper based on the correlation between visceral perception (e.g., visual, auditory, tactile, and other sensory variables), behavior (e.g., player skill level and actual combat experience), reflection (e.g., player acquisition of game culture and information), and game addiction, the following hypotheses are posited:

*H1*: Visceral perception is positively associated with MOBA game addiction.

*H2*: Behavior exhibits a positive association with MOBA game addiction.

*H3*: Reflection demonstrates a positive association with MOBA game addiction.

### Leisure

Leisure is a distinct and subjective mental state, intricately linked to motivation and time (Voss, 1967). When an individual attains a specific psychological state of freedom outside of work, it is considered leisure time. In the early days of the Industrial Revolution, leisure was confined to the “idle class,” exempt from labor-related obligations (Carnevali and Strange, 2014). However, with technological advancements and increased productivity, employers reduced working hours, providing workers with more free time for leisure activities. The evolving work-leisure relationship prompted the study of Leisure Theory (LT) to adopt a multidisciplinary approach (Spracklen et al., 2017). While recent studies on leisure have embraced interdisciplinary fields such as sociology, geography, and culturology, scholars from each discipline have contributed varying definitions of leisure to conceptualize the concept and construct theoretical models. Despite increased scholarship to distinguish the relationship between leisure and other disciplines, this study introduces a theoretical model within leisure activities, specifically focusing on MOBA.

Digital technology has transformed the social dynamics of leisure activities, creating a leisure space intertwined with social attributes (Rojas de Francisco et al., 2016). Social media, recognized as a leisure activity by an increasing number of young people, plays a significant role in users’ lives (Andreassen, 2015; Kuss and Griffiths, 2017; Sharma et al., 2020). Authors have explored the impact of social media on individual users’ self-reflection and emotions within the framework of LT (Sharma et al., 2020). Variables such as age, education level, income, gender, occupation, and social class are commonly assessed for their influence on social media use (Whitfield et al., 2020). However, some studies emphasize social media’s role in enabling individuals to connect without constraints of time and space, contributing to social addiction (Kuss and Griffiths, 2017).

Gaming, identified as a new form of social media, serves as an entertainment platform abundant with leisure resources (Javier Cabeza-Ramirez et al., 2021), boasting an estimated two billion players globally (Matuszewski et al., 2020). In the virtual gaming world, individuals can momentarily escape reality, alleviating pressures from real life and making it a popular leisure activity for various age groups (Tobon et al., 2020). Consequently, studies on gaming culture have gained attention, highlighting the close relationship between leisure and social activities (Andreassen et al., 2017). Engaging in screen-based leisure is significantly linked to an individual’s physical activity, body mass index, social situation, and emotions (Li et al., 2020). Thus, this study investigates whether leisure influences gamers’ emotional experiences, potentially leading to addiction through leisure variables such as decompression, entertainment, and virtual engagement. The following hypotheses are posited:

*H4a*: Leisure is positively associated with gamers’ visceral perception of playing MOBA games.

*H4b*: Leisure is positively associated with gamers’ behavior while playing MOBA games.

*H4c*: Leisure is positively associated with gamers’ reflection on playing MOBA games.

### Uses and gratification

User and Gratification Theory (UGT) is a mass communication theory employed to investigate societal behaviors and motives in the pursuit of specific media to fulfill their needs (Whiting and Williams, 2013). A multidimensional analysis often examines basic needs, individual differences, and cultural backgrounds to understand an individual’s interactions and intentions to use specific media (Rosengren, 1974). With the evolution of new and digital media, UGT is now applied to elucidate why and how individuals choose social media platforms like Instagram, Snapchat, Facebook, and Twitter (Sheldon and Bryant, 2016; Alhabash and Ma, 2017; Phua et al., 2017), games such as League of Legends (LOL) and Pokémon Go (Macedo and da Cunha, 2017; Hamari et al., 2019; Jang and Liu, 2019; Bueno et al., 2020), or video streaming sites like YouTube and Vimeo (Ruggiero, 2000; Khan, 2017; Golob et al., 2021). Not surprisingly, diverse outcomes are expected due to media use being based on individual differences (Greenberg et al., 2010).

UGT has recently been introduced to studies on game behavior and motivation, exploring factors that attract players to video games (Hamari et al., 2019; Jang and Liu, 2019; Bueno et al., 2020; Patzer et al., 2020). Previous research has established a connection between game gratification and motivation, as well as the intention of players’ sustained involvement in these games (Jang and Liu, 2019) and the level of satisfaction felt while playing (Phan et al., 2016). Using UGT, researchers have analyzed the relationship between player gratification and in-game consumption behavior (Ghazali et al., 2019; Hamari et al., 2019), in-game consumption behavior (Wang and Yu, 2017), as well as the connection between player viscosity and experience (Bueno et al., 2020).

If a player has a positive experience during the game, they will be focused, curious, interested, and willing to continue the gaming experience (Choi and Kim, 2004). This may lead to the possibility of further involvement and continuation in the game. Some researchers maintain there is a significant correlation between gratification and players’ motivation to continuously play these games (Abbasi et al., 2017). The motivation for game continuity and players’ long-term gratification experience with the game are the main reasons most players become addicted (Hamari and Keronen, 2017). A recent report by the World Health Organization highlighted the high risks of negative effects of game addiction on various aspects of players’ personal lives, including family, social, professional, and other related areas (Gros et al., 2020). Players’ curiosity about the game, their achievements, and gratification can influence their gaming experience, potentially leading to game addiction. Therefore, this study advances the following assumptions:

*H5a*: Gratification positively influences players’ visceral perception of playing MOBA games.

*H5b*: Gratification positively influences players’ behavior in playing MOBA games.

*H5c*: Gratification positively influences players’ reflection on playing MOBA games.

**Figure 1:**
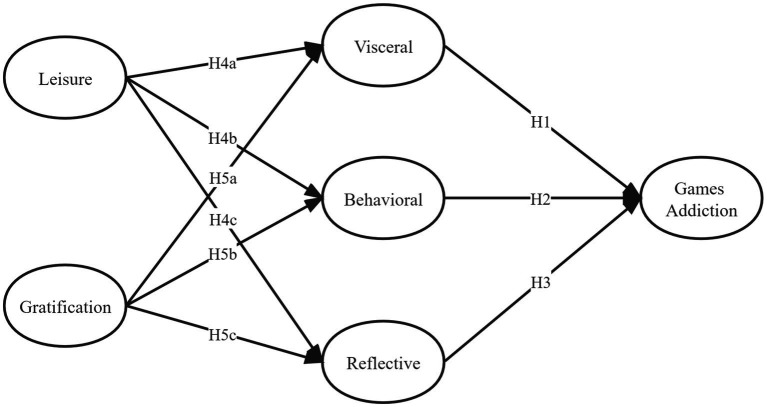
The emotional MOBA game addiction model.

Building upon the review of these theoretical frameworks, it is proposed that leisure and gratification may exert a positive influence on the visceral perception, behavior, and reflection of MOBA game players. Furthermore, a consistently positive gaming experience is identified as the crucial factor in fostering prolonged engagement among MOBA game players. In light of the aforementioned assumptions, an exploratory research model (refer to Figure 1) for a emotional analysis of MOBA game addiction has been formulated.

## Research methodology

### Participants

A total of 298 samples were systematically gathered using a non-probabilistic sampling approach from 24th Nov. 2021 to 24th Mar. 2022 in China. Inclusion criteria encompassed individuals expressing a willingness to volunteer and those engaged in playing MOBA games, specifically League of Legends (LOL) and Honor of Kings (HOK), recognized as the prevailing MOBA games in China (Wirtz, 2022). Additionally, respondents fell within the age bracket of 12 to 40. According to 2017 data from the Nielsen Company, two-thirds of the U.S. population aged 13 and above identified as game players. Notably, 62 participants did not partake in MOBA games and were consequently excluded from the study. The dissemination of online questionnaires transpired randomly through Wenjuanxing, a platform akin to Amazon Mechanical Turk. The conclusive analysis was based on 236 validated questionnaires, equating to a commendable 79.2% response rate among the entire sample pool. It is crucial to note that the act of agreeing to participate in the experiment inherently signified informed consent.

### Questionnaire design

The questionnaire was structured into two sections. The initial segment gathered socio-demographic data, encompassing information such as respondents’ gender, age, education level, employment status, salary, gaming experience, and proficiency. The second section comprised 43 five-point Likert scales, ranging from 1 (totally disagree) to 5 (totally agree). For the emotional analysis of gamers’ addictive behavior, a validated scale for design, adjustment, modification, and optimization was employed. The scales developed by this study are attached in Appendix.

In measuring the average weekly/daily MOBA gaming hours, survey items are adapted from “Usage (UG)” construct from Wu and Holsapple’s (2014) scale.

Visceral perception experience include visual experience and auditory experience. Therefore, survey items are adapted from the following studies: (1) survey items in “Sensory Experience” construct from Abbasi et al.’s (2019, 2021, 2023) measurement models are adapted; (2) survey items in “Sensory & imaginative Immersion” category from Phan et al.’s (2016) GUESS scale are adapted; (3) survey items from Tomlinson et al.’s (2018) BUZZ scale are adapted.

MOBA gamers’ behavioral performance involves both game skills and challenges. Therefore, survey items are adapted from the following studies: (1) survey items in “Behavioral intention to play (BI)” construct from Wu and Holsapple’s (2014) scale are adapted; (2) survey items concerning with skills and challenges from Phan et al.’s (2016) GUESS scale are adapted; (3) survey items in “Perceived behavioral control” construct from Hollebeek et al.’s (2022) measurement model are adapted; (4) survey items concerning with challenges from Bueno et al.’s (2020) scale are adapted.

Gamers’ reflection of the game include both perceptions of the game information and perceptions of the game culture. Therefore, regarding the measurement of gamers’ perception of information, the survey items are adapted from the following studies: (1) survey items in “Cognitive Engagement” construct from Abbasi et al.’s (2023) scale are adapted; (2) the item “I am likely to recommend this game to others.” from Phan et al.’s (2016) GUESS scale is adapted. For measuring cultural cognition, survey items are adapted from following studies: (1) survey items in “Cognitive Engagement” construct from Abbasi et al.’s (2023) scale are adapted; (2) survey items from the “Perceived behavioral control” construct from Hollebeek et al.’s (2022) measurement model are adapted; (3) survey items in “Game Knowledge” construct from Jang and Liu’s (2019) scale are adapted.

Playing games is one of the most popular forms of leisure activities Abbasi et al.’s (2021). Playing games can create escapism and relieve stress for gamers, and immerse themselves in the virtual worlds. A large number of gamers take games as a leisure activity to entertain themselves and spend their free time. Therefore, survey items are adapted from the following studies: (1) survey items concerning escapism and avatars from Abbasi et al.’s (2023) scale are adapted; (2) survey items in “Escapism” construct from Bueno et al.’s (2020) scale are adapted; (3) survey items concerning characters or avatars from Phan et al.’s (2016) GUESS and Segaran et al. (2021) scale are adapted.

Achievement and satisfaction are the two core factors that enhance MOBA gamers’ gratification. Therefore, regarding the measurement of satisfaction, survey items are adapted from the following studies: (1) survey items in “Enjoyment” construct from both Abbasi et al.’s (2023) and Hamari et al.’s (2019) scale are adapted; (2) survey items in “Entertainment” construct from Jang and Liu’s (2019) scale are adapted; (3) survey items in Patzer et al.’s (2020) GUESS Subscales are adapted.

For measurement of MOBA game addiction, survey items are adapted from the following studies: (1) survey items in “Continuance Intention (CI)” construct from Bueno et al.’s (2020) scale are adapted; (2) survey items in “Emotional involvement (EI)” construct from Wu and Holsapple’s (2014) scale are adapted; (3) survey items in “Continuance use intention (CUI)” construct from Jang and Liu’s (2019) scale are adapted.

In summary, addiction factors among MOBA gamers were analyzed in this study across six constructs: Leisure, Gratification, Visceral, Behavioral, Reflective, and Games Addiction. The construct of visceral include visual perception (VP) and auditory perception (AP). The construct of behavioral performance include skill (Sk) and challenge (Ch). The construct of reflective include sharing information (SI) and cultural (Cl) cognition. The construct of gratification include satisfaction (Sat) and achievement (Ach).

### Procedure

The socio-demographic analysis in this study was conducted by Statistical Package for the Social Sciences (SPSS, Version 26). For model evaluation, the Partial Least Squares Structural Equation Modeling (PLS-SEM) was used to assess the relationships between indicators and the measurement models, as well as the structural models. PLS-SEM, known for its capacity to manage complex models and maximize the variance in dependent variables, was selected for its suitability in conducting formative measures on latent variables and fitting the exploratory nature of this theoretical model analysis (Hair et al., 2017; 116). The analysis utilized SmartPLS 4.1.0, involving two key stages:

In Stage 1, four First-order models, Gratification, Reflective, Behavioral and Visceral, were evaluated for measuring structure modeling. As reviewed above, Gratification contains two First-order measurement models, Ac and Sat; Reflective contains two First-order measurement models, Cl and SI; Behavioral contains two First-order measurement models, Ch and Sk; and Visceral contains two First-order measurement models, VP and AP. Thus, in the First-order measurement structural model, we evaluated Factor Loading (FL), Cronbach’s Alpha (Cα), Composite Reliability (CR) value, Average Variance Extracted (AVE) value, and Variance Inflation Factor (VIF) value, intending to assess the reliability and convergent validity of First-order measurement structural models.

In Stage 2, this study constructed a Second-order structural model consisting of six constructs: Leisure, Gratification, Visceral, Behavioral, Reflective, and Games Addiction. First, the reliability and convergent validity of its measurement constructs were assessed by the same assessment method as in Stage 1. Second, the indirect and overall effects between each construct were evaluated, assessing reflective constructs to determine the significance of the relationships posited in the hypotheses.

## Findings and results

### Demographic analysis

Demographic analysis revealed seven key variables among the respondents: gender, age, education, financial status, employment status, MOBA gaming skills, and years of playing MOBA games. The distribution between male (46.2%) and female (53.8%) participants indicated a balanced gender representation in MOBA gaming. The age range of 18 to 30 years dominated the sample, comprising 80.9%, with no minors participating. This age distribution aligns with findings from the Pew Research Center (2018) and the Nielsen Company (2017), which highlighted a predominant young adult demographic in gaming, especially among the Z Generation and Millennials (Dimock, 2019; Lim and Parker, 2020). A significant majority of the respondents were unmarried (91.1%) and well-educated, with over 78.0% holding a bachelor’s degree. This educational status correlates with the high percentage of college students (64.0%) in the sample, who generally have more leisure time compared to high school students and working adults. Employees formed the second-largest group at 25.9%. In terms of gaming proficiency, 75% of respondents were high-level players, and a substantial 83.0% had over 3 years of gaming experience. These demographic details are further elaborated in Table 1.

**Table 1:** Socio-demographic characteristics of MOBA gamers (*N* = 236).

Measure	Variable	Frequency	Percentage (%)
Gender	Male	109	46.2
	Female	127	53.8
Age range	12–17	23	9.7
	18–23	149	63.1
	24–29	42	17.8
	≥30	22	9.4
Education	≤ High School	52	22.0
	Undergraduate	159	67.4
	≥ Postgraduate	25	10.6
Marital status	Single	215	91.1
	Married	21	8.9
Employment Status	Student	151	64.0
	Entrepreneur	12	5.1
	Employee	61	25.9
	Management	12	5.0
Skills	Green Hand	7	3.0
	Primary	21	8.9
	Intermediate	66	28.0
	Advanced	45	19.0
	Master	97	41.1
Years of MOBA game experience	1 year	19	8.1
	2 years	21	8.9
	3 years	28	11.9
	4 years	23	9.7
	≥5 years	145	61.4

### Game duration analysis

**Figure 2:**
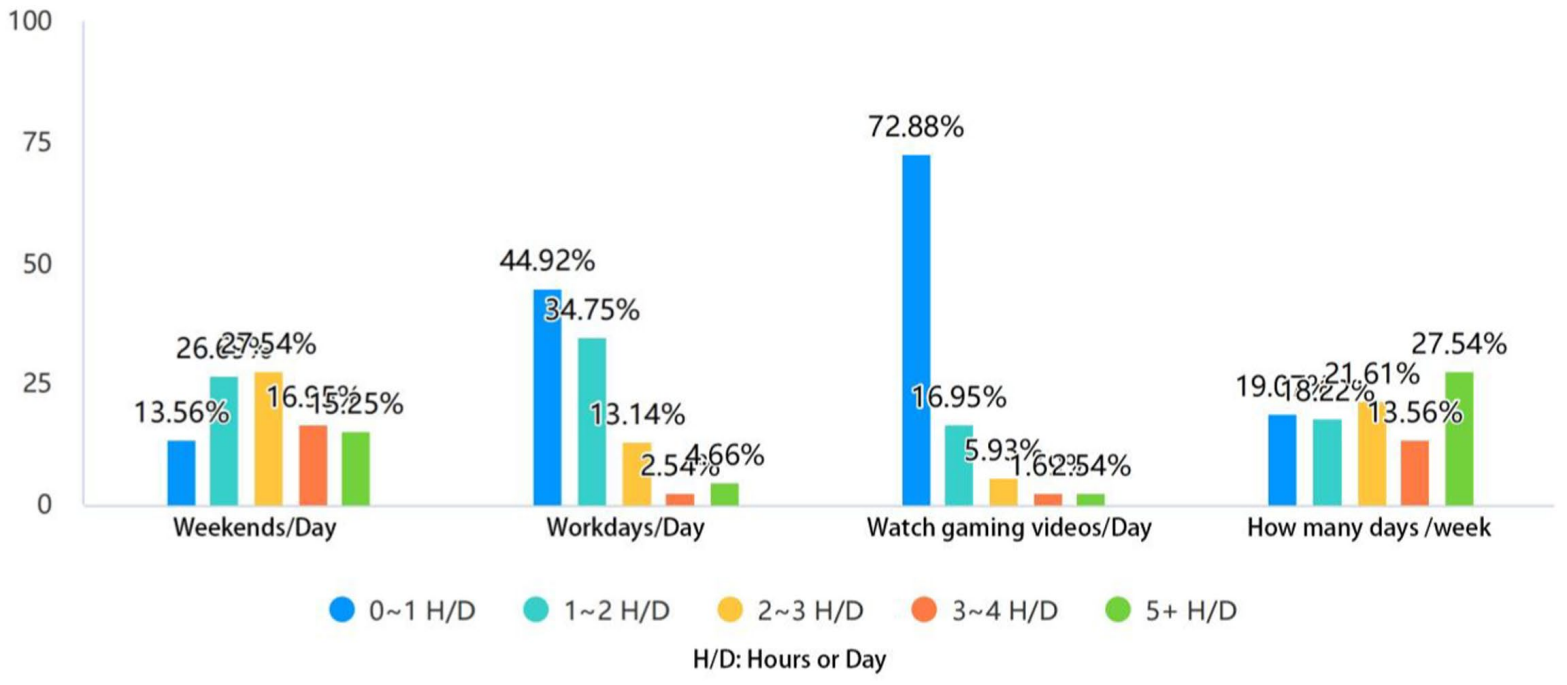
Comparative analysis of MOBA gamers’ game duration (*N* = 236).

The statistics of gamers’ weekly or daily engagement in MOBA games are shown in Figure 2. This study measured the length of time related to gaming activities in gamers’ daily lives from four aspects: (1) the length of time that gamers spend on games per day on weekends; (2) the length of time that gamers spend on games per day on weekdays; (3) the length of time that gamers spend on watching videos related to MOBA games per day; and (4) the days that gamers spend on games every week.

Table 2 shows that among 236 samples, the proportion of gamers playing MOBA games for more than 2 h (2 ~ 3 h, 3 ~ 4 h, >5 h) on weekends (27.54, 16.95, 15.25%) is higher than that on weekdays (13.14, 2.54, 4.66%), with a distinct increase on weekends. The proportion of gamers playing MOBA games for less than 2 h (<1 h, 1-2 h) on weekends (13.56, 26.69%) is smaller than that on weekdays (44.92, 34.75%). It indicates that gamers are more likely to get engaged in MOBA games with longer leisure time on weekends.

**Table 2:** Statistical analysis of MOBA gamers’ game duration (*N* = 236).

Items	<1 H/D	1 ~ 2 H/D	2 ~ 3 H/D	3 ~ 4 H/D	>5 H/D	Mean
How many hours did you spend on playing MOBA game per day on weekends?	32 (13.56%)	63 (26.69%)	65 (27.54%)	40 (16.95%)	36 (15.25%)	2.94
How many hours did you spend on playing MOBA game per day on workdays?	106 (44.92%)	82 (34.75%)	31 (13.14%)	6 (2.54%)	11 (4.66%)	1.87
How many hours did you spend on watching MOBA game videos per day?	172 (72.88%)	40 (16.95%)	14 (5.93%)	4 (1.69%)	6 (2.54%)	1.44
How many days did you spend on playing MOBA games per week?	45 (19.07%)	43 (18.22%)	51 (21.61%)	32 (13.56%)	65 (27.54%)	3.12
Total	355 (37.61%)	228 (24.15%)	161 (17.06%)	82 (8.69%)	118 (12.5%)	2.34

Second, the average length of time gamers spend on watching MOBA online game videos in their daily life is around 1 ~ 2 h (Mean = 1.44, see Table 2), of which 89.83% of gamers watch MOBA online game videos for 0 ~ 2 h per day. It indicates that gamers also spend their leisure time watching MOBA online game videos when they are not engaged in MOBA games.

Third, as shown in Table 2, 58.90% of gamers spend 3 days in average playing MOBA games per week (Mean = 3.12). 80.30% of the gamers spend up to 3 h per day (Mean = 2.41) in playing MOBA games, indicating that gamers have developed the habit of playing MOBA games to some extent. This finding is consistent with the study by De Grove et al. (2016), who found that gamers’ behavior of repeated engagement in games contributed to the development of personal habits.

### Stage 1: first-order reflective models analysis

The convergence effectiveness of the conceptual model was evaluated using CR, Cα, AVE, and VIF. Abbasi et al. (2021) and Hair et al. (2019) established that for the structural model to pass reliability and validity tests, Cα, Rho A, and CR values should exceed 0.70, AVE should be greater than 0.50, while VIF should be lower than 5. As shown in Table 3, survey items (15 items) with FL value lower than 0.70 are deleted, leaving a total of 28 question items in the four First-order reflective models of Gratification, Reflective, Behavioral and Visceral with FL value (0.707 ~ 0.954) greater than 0.70.

**Table 3:** Reliability and validity test of the first-order reflective models (*N* = 236).

Constructs		Items	FL	STDEV	*T*-value	*p*-value	VIF	Cα	CR	AVE
Gratification (*α* = 0.885; AVE = 0.653; CR = 0.886; *p* = 0.000***)	AC	AC2	0.920	0.014	65.118	0.000	3.637	0.880	0.926	0.808
		AC3	0.925	0.012	75.060	0.000	3.750			
		AC4	0.848	0.025	33.727	0.000	2.168			
	Sat	Sat1	0.917	0.014	66.813	0.000	2.878	0.854	0.858	0.775
		Sat2	0.895	0.019	48.056	0.000	2.738			
		Sat3	0.826	0.038	21.629	0.000	1.816			
Reflective (*α* = 0.891; AVE = 0.647; CR = 0.891; *p* = 0.000***)	Cl	Cl1	0.929	0.017	56.129	0.000	3.629	0.928	0.928	0.874
		Cl2	0.954	0.009	109.520	0.000	4.964			
		Cl3	0.922	0.018	52.366	0.000	3.445			
	SI	SI1	0.909	0.018	49.847	0.000	2.814	0.916	0.916	0.856
		SI2	0.948	0.009	109.906	0.000	4.502			
		SI3	0.918	0.018	49.846	0.000	3.380			
Visceral (*α* = 0.886; AVE = 0.612; CR = 0.869; *p* = 0.000***)	AP	AP1	0.895	0.017	52.317	0.000	2.859	0.862	0.869	0.710
		AP2	0.879	0.021	41.261	0.000	2.686			
		AP3	0.786	0.037	20.993	0.000	1.822			
		AP4	0.804	0.030	26.617	0.000	1.802			
	VP	VP1	0.828	0.023	35.860	0.000	2.290	0.787	0.794	0.612
		VP2	0.819	0.023	35.397	0.000	2.045			
		VP3	0.707	0.058	12.286	0.000	1.504			
		VP4	0.769	0.035	22.033	0.000	1.562			
Behavioral (*α* = 0.885; AVE = 0.557; CR = 0.889; *p* = 0.000***)	Ch	Ch1	0.789	0.035	22.741	0.000	1.769	0.851	0.855	0.692
		Ch2	0.842	0.028	29.685	0.000	2.107			
		Ch3	0.826	0.030	27.614	0.000	2.004			
		Ch4	0.869	0.021	42.079	0.000	2.237			
	Sk	Sk1	0.893	0.020	45.505	0.000	2.894	0.914	0.914	0.795
		Sk2	0.895	0.020	44.271	0.000	2.996			
		Sk3	0.915	0.014	64.889	0.000	3.503			
		Sk4	0.862	0.024	35.658	0.000	2.367			

Ac, Achievement; Sat, Satisfaction; Cl, Culture; SI, Share Information; AP, Audit Perception; VP, Visual Perception; Ch, Challenge; Sk, Skill; FL, Factor Loading; STDEV, T-statistic; Cα, Cronbach’s Alpha; CR, Composite Reliability; AVE, Average Variance Extracted; VIF, Variance Inflation Factor.

With 15 survey items deleted, the Cα value (0.787 ~ 0.928) and CR value (0.794 ~ 0.928) of the eight constructs (AC, Sat, Cl, SI, AP, VP, Ch and Sk) were greater than 0.70, and the VIF value (1.504 ~ 4.964) was less than 5, which indicated that survey items in each First-order model had good reliability. Second, the AVE value (0.612 ~ 0.874) of each construct, which is greater than 0.50, indicates that these survey items and constructs have good convergent validity. Third, the Cα (0.885 ~ 0.891), AVE (0.557 ~ 0.653) and CR (0.869 ~ 0.891) values of the four constructs Gratification, Reflective, Behavioral and Visceral passed the test and the *p* values was less than 0.001, indicating that these four First-order models passed the reliability test.

In terms of differential validity assessment, Henseler et al. (2015) proposed a new assessment method, heterotrait-monotrait (HTMT), to measure the correlation between different constructs and thus assess the differential validity between each construct. In this case, an HTMT ratio below 0.85 indicates good discriminant validity between the constructs (Hair et al., 2019). In this study, the four First-order models are tested for differential validity separately, and the results are shown in Table 4, where the HTMT ratios between the constructs are significantly lower than 0.85, indicating that the four reflective First-order models of Gratification, Reflective, Behavioral and Visceral all have good differential validity.

**Table 4:** Heterotrait-montrait (*N* = 236).

Constructs		HTMT value
Gratification	Sat < −> Ac	0.699
Reflective	SI < -> Cl	0.538
Behavioral	VP < -> AP	0.839
Visceral	Sk < −> Ch	0.558

### Stage 2: second-order reflective models analysis

In the Second-order reflective models analysis, the Partial Least Squares (PLS) algorithm was employed to assess the structural model’s reliability and validity. The maximum sub-sample size was set at 5000, and confidence intervals (CI) were calculated using the Percentile Bootstrapping method. The testing was conducted using a two-tailed approach, with a significance level set at *p* < 0.05. Table 5 displays FL and VIF value for each item, and constructs FL, mean, standard STDEV, *t*-values, *p*-values, CR, Cα, and AVE values for Leisure, Gratification, Visceral, Behavioral, Reflective and Games addiction.

**Table 5:** Reliability and validity test of second-order measurement model.

Construct	Items	I-FL	VIF	C-FL	M	S-STDEV	*T*	*p*	CR	Cα	AVE	CI
Leisure	VW1	0.892	2.292	0.822	0.821	0.022	37.506	0.000	0.933	0.892	0.822	[0.776–0.861]
	VW2	0.934	3.501									
	VW3	0.893	2.811									
Gratification	LV scores Ac	0.883	1.580	0.803	0.803	0.022	35.707	0.000	0.890	0.755	0.803	[0.757–0.847]
	LV scores Sat	0.908	1.580									
Visceral	LV scores AP	0.925	1.936	0.848	0.847	0.021	39.572	0.000	0.917	0.820	0.848	[0.803–0.886]
	LV scores VP	0.916	1.936									
Behavioral	LV scores Sk	0.822	1.324	0.744	0.744	0.030	24.958	0.000	0.853	0.662	0.744	[0.681–0.799]
	LV scores Ch	0.902	1.324									
Reflective	LV scores Cl	0.853	1.328	0.748	0.747	0.031	23.977	0.000	0.856	0.664	0.748	[0.684–0.805]
	LV scores SI	0.877	1.328									
Games Addicton	GA1	0.949	2.662	0.895	0.895	0.017	51.582	0.000	0.945	0.883	0.895	[0.858–0.926]
	GA2	0.943	2.662									

I-FL, Items Factor Loading; VIF, Variance Inflation Factor; C-FL, Construct Factor Loading; M, Mean; S-STDEV, Standard deviation (STDEV); T, t-value; P, p-value; CR, Composite Reliability; Cα, Cronbach’s Alpha; AVE, Average Variance Extracted; CI, Confidence Intervals.

The findings indicate that I-FL values (0.853–0.949) for each item are greater than 0.70, and VIF values (1.324–3.501) are less than 5, indicating good reliability of the measurement constructs. C-FL (0.744–0.895), CR (0.853–0.945), and Cα (0.662–0.895) values are greater than 0.70 for each of the six constructs. AVE values (0.744–0.895) greater than the minimum threshold of 0.50 and the *p*-values are less than 0.001. These findings confirm that the Second-order structural model successfully meet the criteria for reliability and validity testing.

The model’s uniqueness was affirmed through a discriminant validity test, an essential criterion for verifying reflective structural models as noted by Hair et al. (2017). Latent variable correlation (Fornell-Larker, F-L) is another way to test the discriminant validity, where the value of the square root of AVE should be greater than the correlation coefficient between each constructs (Hair et al., 2017, 2019, 2022). In other words, the explanation of observed variables by latent variables should be better than the explanation of other latent variables in order to pass the discriminant validity test.

The findings, presented in Table 6, show that HTMT value of Gratification <− > Behavioral, Reflective <− > Behavioral, and Reflective <− > Gratification are 0.937, 0.955, and 0.904, respectively, which are all greater than 0.85, indicating that internal correlation exist among constructs of Behavioral, Gratification and Reflective. The HTMT values of the other constructs are less than 0.85, indicating that certain internal correlation exist among some constructs in the model. Therefore, this study is further tested by F-L. It is also found that the value of the square root of AVE between each construct is greater than the correlation coefficient between each construct, which passed differential validity. It indicates differential validity is supported, displaying good internal consistency between each constructs in the model of this study.

**Table 6:** Discriminant validity Fornell-Larcker and HTMT values.

	Behavioral	Games Addicton	Gratification	Leisure	Reflective	Visceral
Behavioral	**0.863**					
Games Addicton	0.484 (0.633)	**0.946**				
Gratification	0.680 (*0.937*)	0.454 (0.558)	**0.896**			
Leisure	0.425 (0.531)	0.416 (0.469)	0.476 (0.572)	**0.907**		
Reflective	0.651 (*0.955*)	0.489 (0.636)	0.639 (*0.904*)	0.645 (0.835)	**0.865**	
Visceral	0.489 (0.670)	0.423 (0.494)	0.506 (0.639)	0.337 (0.389)	0.415 (0.565)	**0.921**

Values in bold are AVE square root values; Fornell-Larcker before parentheses; HTMT values inside parentheses; Underlined italicized values indicate HTMT values above 0.90.

**Figure 3:**
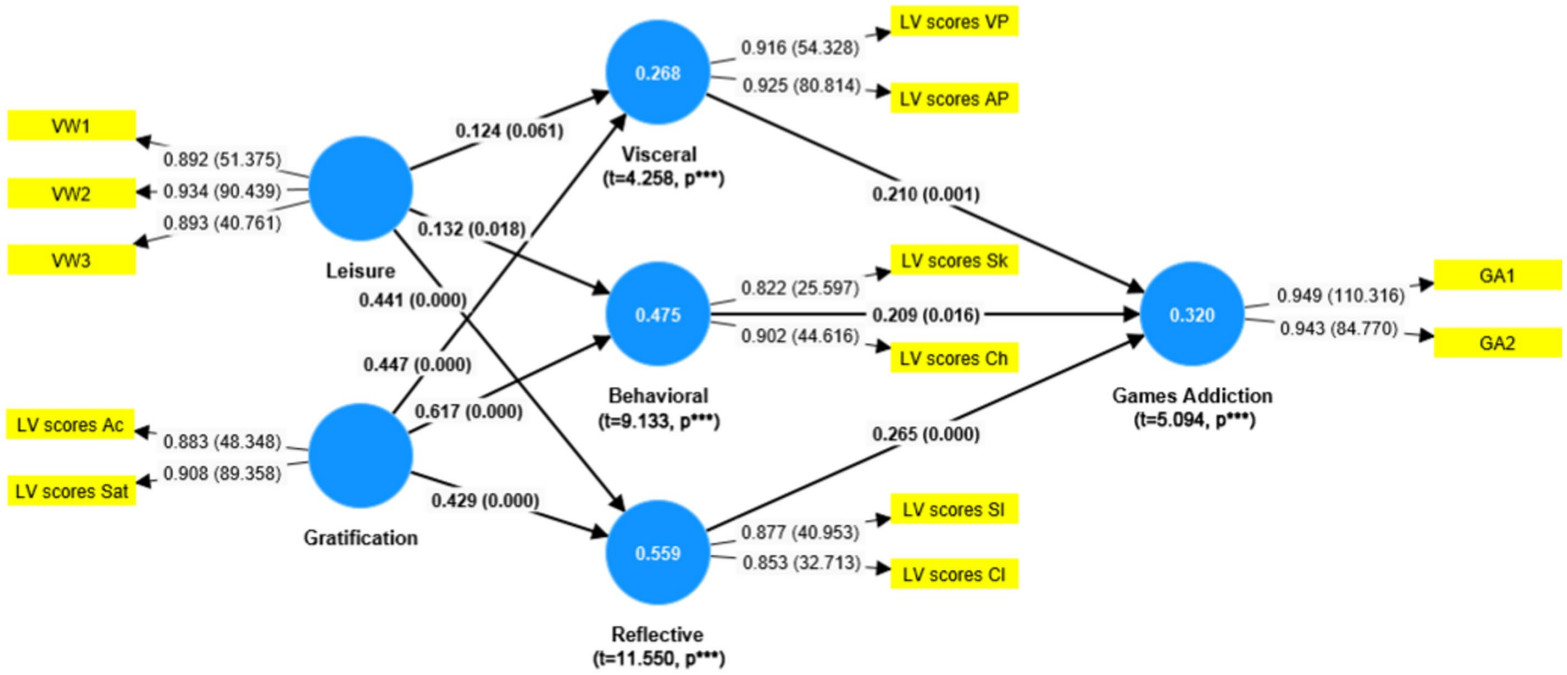
Validation model of the study model.

In the structural model and hypothesis testing, this study utilized the Partial Least Squares (PLS) automatic estimation method for measuring structural models and verifying hypotheses. The final verification model is depicted in Figure 3, and the total effects are detailed in Table 7. The *t*-values for H1, H2, and H3 were 3.391, 2.415, and 3.541, respectively, with corresponding *p*-values of <0.01, < 0.05, and < 0.001, respectively, and positive path coefficients. These results support H1, H2, and H3, indicating that visceral, behavioral, and reflective aspects positively influence over-engagement in MOBA games by gamers.

**Table 7:** Total effect of the verification model of the hypotheses.

Hypotheses	FL	S-STDEV	*T*	*p*	Result
*H1*: Visceral> Games Addiction	0.210	0.062	3.391	0.001**	Supported
*H2*: Behavioral> Games Addiction	0.209	0.087	2.415	0.016*	Supported
*H3*: Reflection> Games Addiction	0.265	0.075	3.541	0.000***	Supported
*H4a*: Leisure> Visceral	0.124	0.066	1.876	0.061	Unsupported
*H4b*: Leisure> Behavioral	0.132	0.056	2.362	0.018*	Supported
*H4c*: Leisure> Reflection	0.441	0.052	8.471	0.000***	Supported
*H5a*: Gratification> Visceral	0.447	0.068	6.569	0.000***	Supported
*H5b*: Gratification> Behavioral	0.617	0.048	12.747	0.000***	Supported
*H5c*: Gratification> Reflection	0.429	0.054	7.928	0.000***	Supported
Gratification> Games Addiction	0.171	0.039	4.364	0.000***	Supported
Leisure> Games Addiction	0.337	0.044	7.735	0.000***	Supported

*p < 0.05, **p < 0.01, ***p < 0.001.

The *t*-values for H4a is 1.876, with *p*-value of 0.061, greater than a 0.05 threshold. This leads to the rejection of H4a, suggesting that leisure does not significantly influence gamers’ visceral perception in MOBA games. However, H4b and H4c, with *t*-values of 8.471 and 6.569 respectively, and *p*-values were less than 0.05 and 0.001 respectively, are supported, indicating a significant influence of leisure on gamers’ behavioral and reflective states. Additionally, the indirect effect of leisure on MOBA game engagement, with a *t*-value of 7.735 and a *p*-value less than 0.001, suggests a significant overall impact of leisure on game engagement.

For H5a-c, the *t*-values were 6.569, 12.747, and 7.928, respectively, with all *p*-values <0.001 and positive path coefficients, validating these hypotheses. These findings indicate that gratification significantly influences gamers’ visceral perception, behavior, and reflection. Moreover, the *t*-value for the indirect effect of gratification on MOBA game engagement was 4.364, with a *p*-value <0.001, demonstrating a substantial overall impact of gratification on engagement in MOBA games.

### Model quality assessment

PLS-SEM model quality assessment includes the coefficient of determination (R^2^), which is used to evaluate the explanatory and predictive power of the model. The value of R^2^ ranges from 0 to 1, with higher values indicating greater explanatory power. The criteria for assessing the *R*^2^ value are inconsistent across different disciplines and research areas (Hair et al., 2022).

The *R*^2^ of the theoretical model of MOBA addiction was assessed by SmartPLS 4.1.0 based on Bootstrapping algorithm. The results are shown in Table 8. The effects of Leisure and Gratification on Visceral (*T* = 4.258, *p* < 0.001), Behavioral (*T* = 9.133, *p* < 0.001), and Reflective (*T* = 11.550, *p* < 0.001) constructs were 26.8, 47.5, and 55.9%, respectively. The explanatory power of Visceral, Behavioral, and Reflective for Games Addiction was 32.0%. It shows that the theoretical model of this study has good quality.

**Table 8:** *R*^2^ quality assessment.

	O	M	STDEV	*T*	*p*	CI [25–95%]
Behavioral	0.475	0.484	0.052	9.133	0.000***	[0.379–0.583]
Games Addicton	0.320	0.332	0.063	5.094	0.000***	[0.209–0.454]
Reflective	0.559	0.565	0.048	11.550	0.000***	[0.469–0.659]
Visceral	0.268	0.276	0.063	4.258	0.000***	[0.157–0.401]

O, Original sample; M, Sample mean; S-STDEV, Standard deviation; T, T statistics (|O/STDEV|); *p*, *p* values; CI, Confidence Intervals.

## Discussion

This study aimed to explore the moderating roles of visceral perception, behavior, and reflection in MOBA gamers, relating to the LT, UGT, and EDT frameworks, and to identify factors contributing to game addiction. Although nine hypotheses were formulated to develop a theoretical model, not all received empirical support.

In Stage 1 the First-order reflective models analysis revealed that MOBA gamers’ visual and audio-visual experiences elicit strong emotional responses, significantly influencing their excessive game involvement. Nuyens et al. (2016) suggested that impulsive and stress reactions from intense game participation might be key psychological factors. Enhanced accessibility of games via smartphones (Chamarro et al., 2020) provides increased playing opportunities, but the MOBA interface’s limited size on mobile devices might diminish sensory experiences. The PLS-SEM model’s validity test showed tactile perception’s dimension load coefficient below 0.70, similarly affected by the screen’s size in some visual perception constructs. However, gamers reported that sound effects and background music in MOBA games greatly enhance the experience. They agreed that muted sound effects would render the game uninteresting. Consequently, all auditory perception dimensions’ load coefficients aligned with reliability and validity standards. Thus, auditory experience emerged as a primary factor in gamers’ MOBA addiction, followed by visual and tactile experiences. Abbasi et al. (2017) corroborated that MOBA games’ audio-visual feedback and stimulation intensify gamers’ sensory experiences, bolstering their intention to continue playing. Mason (2017) previously demonstrated how audio-visual and tactile elements could enhance the gaming experience and promote gamers’ continued engagement.

Secondly, gamers’ proficiency in MOBA control skills and resulting behaviors contributed to their excessive involvement, partly due to the challenge of increasing game difficulty, aligning with findings from Ghazali et al. (2019) and Merikivi et al. (2017). Furthermore, gamers’ deep understanding of characters’ personalities, stories, and design inspirations, alongside cognitive activities like sharing battle experiences and staying updated with game developments, significantly drove their over-engagement. This behavioral and cultural reflection in gaming, a novel finding in the field, was positively correlated with excessive game involvement. Most gamers preferred playing with friends, demonstrating effective socialization skills during gameplay (Chamarro et al., 2020). They also actively engaged with the game’s backstory and character lore, and eagerly gathered the latest game strategies and updates (Wang et al., 2023), indicating a significant and positive relationship between game-related gratification and gamers’ active information-seeking behavior.

Thirdly, emotional involvement in an activity intensifies and prolongs behavior (Mudie, 2003; Ghazali et al., 2019), a concept validated by our findings. Visceral perception, behavior, and reflection all positively influenced gamers’ excessive involvement in MOBA games. MOBA’s visual, auditory, and tactile elements stimulate gamers’ senses, with emotional feedback like rewards and punishments after game outcomes significantly affecting their neurological and physiological responses (Arbeau et al., 2020). This not only heightened gamers’ involvement but also extended their playing time and frequency. Leisure and gratification were identified as key factors promoting gamers’ sensory engagement and gameplay behavior.

Fourthly, while leisure activities did not significantly influence gamers’ visceral perception, they impacted their behavior and reflection on the game. The influence of leisure on gaming behavior is supported by relevant studies. Király et al. (2017, 2022) argued that leisure activities played a beneficial role for gamers’ escapist motivations behaviors. Van Rooij and Nijkamp (2018) employed leisure-time use/diary analysis to understand gamers behavior within the virtual context. He also believed problematic gaming behavior did not happen in a vacuum; in other words, gaming always displaces other activities. Leisure activities contribute to people’s basic needs and growth needs (Joseph Sirgy et al., 2018). Growth needs include cognition on symbolism, esthetics, and morality. This finding supports the conclusion that leisure has a positive effect on gamers’ cognition in this study. In addition, the overall effect mentioned above indicate that leisure is positively correlated with their intention to continue playing. Furthermore, the sense of gratification obtained from playing MOBA games positively correlated with their intention to continue playing.

## Conclusion and limitation

Drawing on UGT, LT, and EDT, this paper proposes a theoretical model for understanding engagement in MOBA games. It synthesizes existing literature, focusing on the nexus between gamers’ emotions and behaviors (Fikkers et al., 2016) and the interplay between emotional drivers and excessive involvement in MOBA games (Nuyens et al., 2016). The model examines how leisure, uses and gratifications, and players’ emotional experiences-encompassing visceral perception, behavior, and reflection-influence their propensity for over-engagement in MOBA games. This research extends the range of variables known to impact MOBA game engagement and underscores the comprehensive nature of the proposed model.

The emotional MOBA game addiction model underwent rigorous evaluation, successfully meeting criteria for reliability and validity, including CR, Cα, Rho A, and AVE measures. Additionally, it passed the HTMT test for discriminant validity, affirming its structural distinctiveness and overall effect. The analysis revealed a positive correlation between personal visceral perceptions and reflections with over-engagement in MOBA games. While personal behaviors had an ambiguous effect on over-engagement, leisure and gratification significantly increased the likelihood of excessive involvement. Notably, the correlation between leisure and players’ reflection was insignificant, predominantly reflected in the behavior of experienced players engaging in MOBA games primarily for leisure and entertainment. These findings suggest that players often engage in games during their busiest times, contradicting the notion of idle gaming.

In summary, this study expands upon existing theoretical frameworks that address MOBA game motivation and addiction, guided by emotional, leisure, and uses and gratification theories. It also offers a theoretical foundation for future research on game reflection, behavior, and over-engagement in MOBA games. Considering the proliferation of mobile technologies like smartphones and smartwatches, further research is warranted to explore their impact on MOBA gaming addiction. Moreover, reconceptualizing gaming as more than a leisure pursuit warrants additional investigation in academic discourse. The theoretical model provides certain theoretical references for further exploration of addition to online video games as well as online electronic devices, etc., and has certain applicability to the research in the field of related areas as Internet Addiction.

However, this study still has certain limitations. From theoretical perspective, this study does not consider the impact of negative behaviors such as the impact on gamers’ emotion brought by aggressive language when exploring the impact of MOBA games on gamers’ behaviors. Previous studies on the influence of leisure activities on gamers’ behavior can support the conclusions of this study. However, the influence of leisure on gamers’ cognition lacks supporting evidence. Secondly, when measuring MOBA gamers’ addiction, we only take the influence of continuance intention on gamers’ addiction into consideration, and experimental studies such as brain stimulation are not taken into account. Therefore, certain limitations still exist. In future studies, the effects of gamers’ negative behavior and the effects of brain stimulation on gamers’ addiction will be fully investigated, and the relationship between leisure and gamers’ cognition will be further explored in order to improve the theoretical model of this study.

## Data availability statement

The original contributions presented in the study are included in the article/Supplementary material, further inquiries can be directed to the corresponding author.

## Ethics statement

The studies involving humans were approved by the Academic and Ethics Committee of the School of Design, Fujian University of Technology. The studies were conducted in accordance with the local legislation and institutional requirements. Written informed consent for participation in this study was provided by the participants' legal guardians/next of kin.

## Author contributions

EH: Conceptualization, Data curation, Formal analysis, Funding acquisition, Methodology, Project administration, Writing – original draft. YX: Conceptualization, Formal analysis, Supervision, Writing – review & editing. XS: Formal analysis, Methodology, Resources, Writing – review & editing.
