# Supplementary material for: Emotional analysis of multiplayer online battle arena games addiction
Source: Front Psychol. 2024 May 9;15:1347949. doi: 10.3389/fpsyg.2024.1347949 (PMC11111968; doi:10.3389/fpsyg.2024.1347949)
Supplement: Supplementary file 2 [file Table_1.pdf]

# APPENDIX

| | Constructs & Items | References |
|---|---|---|
| | ***Visceral*** | |
| *AP* | *AP1* The sound effects in MOBA games could enhance your game experience. | Abbasi et al. (2019, 2021, 2023)<br>Phan et al. (2016) |
| | *AP2* Announcement of game situations will enhance your enthusiasm for MOBA games.<br>*AP3* Announcements from your teammates will enhance your reaction to MOBA games.<br>*AP4* Turning off the game's sound effects will affect your MOBA game experience. | Tomlinson et al. (2018) |
| *VP* | *VP1* The game's visual effects are cool.<br>*VP2* The size of your cell phone screen will affect your viewing experience of the game. | Phan et al. (2016)<br>Abbasi et al. (2019, 2021, 2023) |
| | ***Behavioral*** | |
| *Ch* | *Ch3* You won't stop playing MOBA games until you move to the next level. | Wu & Holsapple (2014)<br>Bueno et al. (2020)<br>Phan et al. (2016)<br>Hollebeek et al. (2022) |
| | *Ch4* Playing MOBA games will make you feel intense and excited. | Bueno et al. (2020)<br>Hollebeek et al. (2022) |
| *Sk* | *Sk1* You have the ability to lead the game. | Hollebeek et al. (2022)<br>Phan et al. (2016)<br>Wu & Holsapple (2014) |
| | *Sk2* You can have a global view of the field through the mini-map.<br>*Sk3* You have the ability to adapt to the situation when your team is fighting. | Wu & Holsapple (2014) |
| | *Sk4* You have multi-key or multi-finger capability. | Wu & Holsapple (2014)<br>Hamari et al. (2019) |
| | ***Reflective*** | |
| *SI* | *SI1* You want to know the latest news about new heroes or new skins etc., in MOBA games.<br>*SI2* You will receive the latest news on the MOBA games. | Abbasi et al. (2023) |
| | *SI3* You will share the latest news or information on the MOBA games. | Abbasi et al. (2023)<br>Phan et al. (2016) |
| *Cl* | *Cl1* You know the characters' or heroes' backstory. | Abbasi et al. (2023)<br>Hollebeek et al. (2022) |
| | *Cl2* You will become familiar with more mythological, historical, or social public figures through the MOBA game. | Jang & Liu (2019) |

| | | |
|---|---|---|
| | *Cl3* You can learn more about the traditional culture through the MOBA games. | Abbasi et al. (2023) |
| | ***Leisure*** | |
| *VW* | *VW1* MOBA games allow you to immerse in the virtual world of the games. | Abbasi et al. (2021)<br>Bueno et al. (2020) |
| | *VW2* You can play any role in MOBA games. | Abbasi et al. (2021) |
| | *VW3* You will feel that the characters in MOBA games are your avatars. | Segaran et al. (2021)<br>Phan et al. (2016) |
| | ***Gratification*** | |
| *Ac* | *Ac2* You want to gain more experience value in MOBA games. | Abbasi et al. (2021) |
| | *Ac3* You want to get more medals, honors, props, heroes or skins, etc., in MOBA games. | |
| | *Ac4* You enjoy the good feeling that comes with winning in MOBA games. | Jang & Liu (2019) |
| *Sat* | *Sat1* MOBA games can bring you more satisfaction. | Patzer et al. (2020) |
| | *Sat2* MOBA games can bring you more pleasure. | Hamari et al. (2019) |
| | *Sat3* MOBA games can add color to your life. | |
| | ***Games Addiction*** | |
| *GA* | *GA1* MOBA games can bring you more excitement. | Bueno et al. (2020)<br>Wu & Holsapple ( 2014) |
| | *GA2* The more you become engaged in MOBA games, the more you want to play it. | Bueno et al. (2020)<br>Jang & Liu (2019) |
